# Supplementary material for: A set of multi-entry identification keys to African frugivorous flies (Diptera, Tephritidae)
Source: Zookeys. 2014 Jul 24;(428):97–108. doi: 10.3897/zookeys.428.7366 (PMC4143993; doi:10.3897/zookeys.428.7366)
Supplement: Supplementary material 4 — Key to Capparimyia [file zookeys-428-097-s004.zip › SF4_ZooKeys_key to Capparimyia/lucid_player/help/help/what_is_a_java_applet_.htm]

What is a Java Applet?


# What is a Java Applet?

An applet is a program written in the Java programming language that
can be embedded in an HTML page, in much the same way as images are included.
When you use a Java technology-enabled browser to view a page that contains
an applet, the applet's code is transferred to your system and executed
by the browser's Java Virtual Machine (JVM). The Lucid3 Player is available
as a Java applet. This means users of your key do not need to download
and install a Lucid Player, as was the case with previous versions.
